# Beneficial Effects of Plant Extracts and Bioactive Food Components in Childhood Supplementation

**DOI:** 10.3390/nu13093157

**Published:** 2021-09-10

**Authors:** Hammad Ullah, Anna De Filippis, Alessandra Baldi, Marco Dacrema, Cristina Esposito, Emanuele Ugo Garzarella, Cristina Santarcangelo, Ariyawan Tantipongpiradet, Maria Daglia

**Affiliations:** 1 Department of Pharmacy, University of Naples Federico II, 80131 Naples, Italy; hammad.ullah@unina.it (H.U.); marco.dacrema@unina.it (M.D.); cristina.esposito@unina.it (C.E.); emanueleugo.garzarella@unina.it (E.U.G.); cristina.santarcangelo@unina.it (C.S.); ariyawanps@gmail.com (A.T.); 2 Department of Experimental Medicine, Section of Microbiology and Virology, University of Campania “L. Vanvitelli”, 80138 Naples, Italy; anna.defilippis@unicampania.it; 3 Tefarco Innova, National Inter-University Consortium of Innovative Pharmaceutical Technologies—Parma, 43124 Parma, Italy; alessandra.baldi.alimenti@gmail.com; 4 International Research Center for Food Nutrition and Safety, Jiangsu University, Zhenjiang 212013, China

**Keywords:** childhood disorders, plant extracts, bioactive components, childhood supplementation

## Abstract

The pivotal role of childhood nutrition has always roused a growing interest from the scientific community. Plant extracts and bioactive dietary components play a significant role in the maintenance of human health and wellness, with the potential to modulate risk factors and manage symptoms for a large number of common childhood disorders such as memory impairment, respiratory illnesses, gastrointestinal disorders, metabolic derangements, and pathologies related to the oral cavity. This review is designed to highlight the health benefits of botanical extracts and bioactive dietary components in children as evidenced by clinical trials, considering their safety with regards to childhood sensibilities. The supplementation of children with the herbal extracts or bioactive components mentioned in this review leads to the conclusion that they are useful for treating various ailments, with no serious adverse events being reported. However, for the limited number of investigations specifically focused on the safety of such products in children, time is needed to expand the literature data covering the safety of childhood supplementation with botanical extract and bioactive food components.

## 1. Introduction

The use of dietary supplements worldwide has increased in the last 30 years [1]. Dietary supplements are used in the age group ranging from birth to 18 years of age, by 31% of the population, to improve overall health (41%), maintain health (37%), supplement the diet (23%), prevent health problems (20%), and “boost immunity” (14%) [2]. From data by the World Health Organization (WHO), around 80% of the adult population in developing countries uses plant extracts for their health needs [3,4,5]. Plant extracts are also used for children, although their use must be regulated by the awareness that children differ from adults in terms of physical size, body composition, and physiology. Medicinal plants can be used to treat winter problems in a preventive context, and thus to strengthen the immune system and improve the body’s adaptation to seasonal disturbances, but they can also be used as a treatment for various symptomatic connotations. It is necessary to know how to regulate the use of such supplements according to a child’s body weight to avoid reaching toxic doses [6]. In a German study, 85% of children used one or more herbal supplement products [5]. Another study reported that 16% of Japanese pediatric surgical patients use herbal supplement products [7].

About 9% of newborns, from the first month of life, have been treated with herbal supplements, in particular, for mild neonatal ailments such as flatulence, teething, or colds. The extracts used are based on chamomile, mint, echinacea, fennel, catnip, and anise [8]. The belief that natural herbal products are safe, culturally significant, cheaper than some medical treatment options, and easily accessible, are some of the reasons why these products are being used [9].

Given that the correct use of food supplements is safe, it is important to rely on a trusted pediatrician in order to verify the existence of a real need to take supplements and subsequently to assess the correct dosage and identify the presence of any contraindications (some plant extracts/supplements cannot be used in children) both in situations of simple nutritional support and in conjunction with the intake of drugs for intercurrent or chronic pathological conditions.

The aim of this review is to consider plant extracts and bioactive food components frequently used as food supplements in different age groups (from birth to adolescence) and in specific situations, so as to counter certain health problems. In detail, the target of our work is to identify which health problems in childhood attract the use of herbal supplements, which plant extracts and bioactive food components are more frequently used, and what beneficial effects they possess.

## 2. Methodology

**Figure 1:**
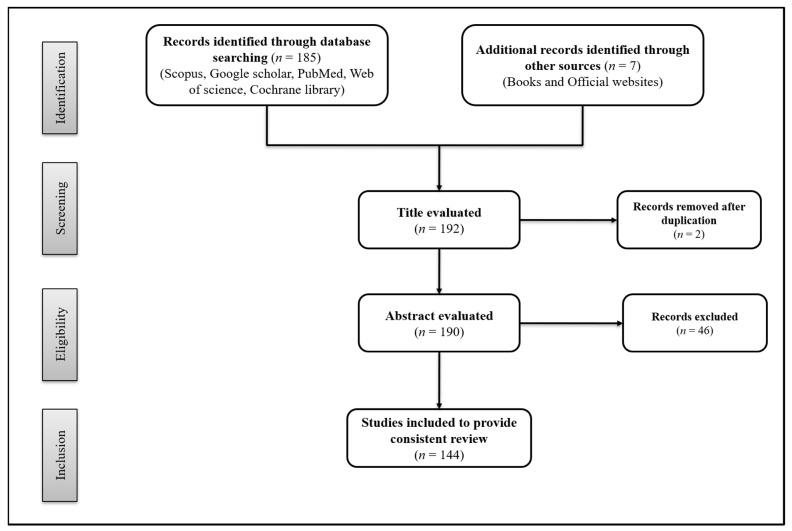
PRISMA flow diagram, showing the process of study selection.

This research covered all studies addressing the beneficial effects of plant extracts used as supplementation against childhood pathologies. For this, we used up-to-date databases, including Pubmed, Web of Science, Scopus, Google Scholar and Cochrane Library. The keywords used in our search were: “childhood supplementation”, “plant extracts”, “childhood pathologies”, “bioactive food” and “bioactive components”. The criteria for selecting articles were “studies reported in English, because of language barriers” and, “clinical studies related to childhood supplementation”. The results returned 185 papers published up to the year 2021. Of these articles, 139 were selected, summarized, and critically discussed so as to provide a consistent review. The main reasons for the exclusion of 48 articles were the fact that they report preclinical studies, are written in a language other than English, and are title duplications. In addition to the 139 articles selected for this review, some books and official websites were also used to provide specific data within the scope of the present study. Figure 1 illustrates the PRISMA flow diagram for the study selection.

In the following sections, the most commonly used plant extracts and bioactive food components are discussed, including their role in childhood supplementation and their beneficial use in childhood pathologies.

## 3. Food Supplements and Childhood Illnesses

**Figure 2:**
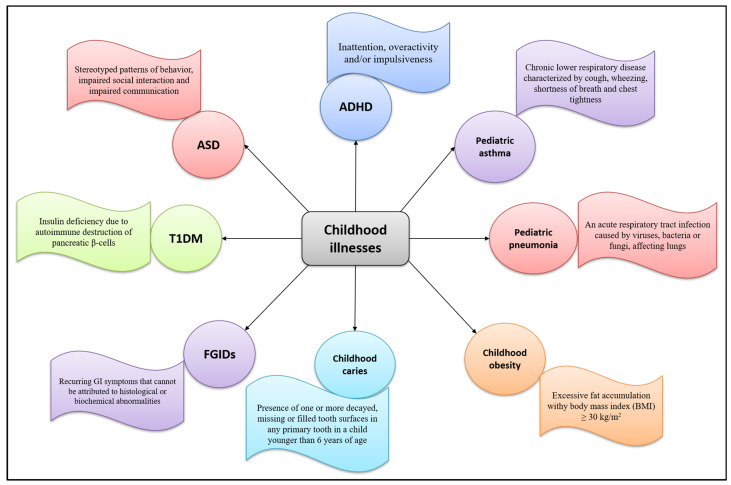
Common childhood illnesses and their defining features. Attention deficit hyperactivity disorder (ADHD), autism spectrum disorder (ASD), Type 1 diabetes mellitus (T1DM), functional gastrointestinal disorders (FGIDs).

A food supplement is a product intended to supplement the diet in particular conditions of deficiency. Children with correct and balanced eating styles rarely need supplements, except for particular cases in the first year of life or in the presence of certain disorders or diseases. During childhood, plant extracts are used to treat symptoms of upper respiratory tract infections, sleeping problems, gastrointestinal disorders, or occasional and common ailments such as cough, cold, and sore throat. Figure 2 summarizes common childhood pathologies and their defining features.

### 3.1. Botanical Extracts

The use of botanical extracts supporting conventional drugs is widespread for common ailments in childhood. The evidence from clinical trials for botanical extract supplementation in children has been summarized in Table 1.

#### 3.1.1. *Melissa officinalis* L.

*Melissa officinalis* L. (lemon balm) is a well-known perennial medicinal plant from the Lamiaceae family, distributed in southern Europe, southern parts of North America, and Asia Minor. It has wide pharmacological effects including purgation, antispasmodic, antibacterial, antifungal, antihistaminic, antioxidant, immune-enhancing effects, and modulating mood and cognitive performance [24,25,26,27]. The plant is quite rich in phenolic acids such as caffeic acid, ferulic acid, gallic acid, and rosmarinic acid, and flavonoids such as flavonols, flavan-3-ols, and flavanones [28]. Other bioactive components present in *M. officinalis* include stilbenes, chromones, anthraquinones and dianthrones [29]. A systematic review conducted by Anheyer and colleagues supported the improvement of attention deficit hyperactivity disorder (ADHD) in children supplemented with *M. officinalis* extract [30]. Katz et al. (2010) carried out a randomized controlled trial to investigate the clinical outcomes of an herbal preparation (*M. officinalis*, *Withania somnifera* (L.) Dunal, *Bacopa monnieri* (L.) Wettst., *Arthrospira platensis* geitl and *Centella asiatica* (L.) Urb.) in children with ADHD [10]. One hundred and twenty children with a mean age of 9.82 years for the treatment group, and 9.36 years for the control group, were recruited in this study and were supplemented with 3 mL of the herbal preparation three times a day in 50–60 mL water. The results showed significant improvement of Test of Variables of Attention (TOVA) scores, attention, cognition, and impulse control in the intervention group when compared to the placebo group.

The usage of lemon balm by mothers in children to cope with sleep problems has also been observed [31]. The effects of *M. officinalis* alone or in combination with *Phytolacca decandra* L. in the treatment of children with possible sleep bruxism have been evaluated in a crossover randomized triple-blinded controlled clinical trial [11]. Children (*n* = 52; mean age = 6.62 years) with sleep bruxism were selected for the study, that included four phases of 30-day treatments (placebo, *M. officinalis*, *P. decandra*, and *M. officinalis* + *P. decandra*), with a washout period of 15 days between the treatments. The results of the trial showed promising results with *M. officinalis* in the treatment of sleep bruxism, as reflected by a decrease in the Visual Analogic Scale (VAS), while the combination of both extracts did not offer any additional benefits.

#### 3.1.2. *Foeniculum vulgare* Mill.

*Foeniculum vulgare* Mill. (fennel) (Apiaceae), is extensively used for flavoring foods and beverages due to its pleasant spicy aroma. Fennel is native to the Circum-Mediterranean area but is cultivated across the globe [32]. The plant and its essential oil have been used in traditional medicine for its carminative, digestive, diuretic, and galactagogue properties [33]. Mothers most commonly supplement their children with fennel to relieve gas pain, constipation, digestive problems, nausea, and vomiting [31]. In a double-blind placebo-controlled study, a mixture of 0.1% fennel oil emulsion and 0.4% polysorbate in water, 5 to 20 mL, was administered to babies diagnosed with infantile colic, four times daily before meals at a maximum of 12 mL/kg/day. The results showed a significant recovery of the colic symptoms in the group treated with fennel, compared with the placebo group [12].

The European Food Safety Association recommended the average intake of fennel in the form of tea as 3 to 5 grams of crumbled fresh plant divided into three doses, which should only be used for less than 7 days and should not be used for children under 4 years of age [34].

#### 3.1.3. *Matricaria chamomilla* L.

*Matricaria chamomilla* L. (German chamomile) is one of the most widely used herbal remedies, belonging to the Asteraceae family and being native to Southern and Eastern Europe. Chamomile is extensively used for its calming effects throughout the world and is included in the pharmacopeia of 26 countries [35,36]. Traditionally, chamomile has been used as a breath freshener, digestive aid, immunity booster, sleep aid, and reliever of allergic symptoms, bronchitis, menstrual problems, and insect bites [36]. The flowers of chamomile are rich in polyphenols such as quercetin, apigenin, luteolin, patuletin, chlorogenic acid, caffeic acid and coumarins [37,38,39].

In a randomized controlled trial, children with infantile colic were treated with a mixture of the standardized extract of *M. chamomilla*, *M. officinalis,* and tyndallized *Lactobacillus acidophilus* (HA122) or *Lactobacillus reuteri* (DSM 17938) for 28 days. One hundred and seventy-six patients completed the study, and the mixture of *M. chamomilla,* and *M. officinalis* with tyndallized *L. acidophilus* (HA122) or *L. reuteri* (DSM 17938) was observed to be most effective in relieving the symptoms of infantile colic as reflected by a significant decrease in mean daily crying time [13]. Javid et al. (2019) treated 46 children (7–12 years old) diagnosed with intermittent asthma, with an herbal mixture including *M. chamomilla*, *Althaea officinalis* L., *Hyssopus officinalis* L., *Malva sylvestris* L., *Adiantum capillus-veneris* L., *Ziziphus jujube* Mill. and *Glycyrrhiza glabra* L. for 5 days [14]. The result reflected a significant reduction in the severity of cough and nighttime awakening in the treatment group, however, no significant improvement of wheezing, respiratory distress, tachypnea, peak expiratory flow rate, asthma exacerbations, outpatient visits, oral prednisolone or β-agonists, and hospitalization was seen.

#### 3.1.4. *Boswellia serrata* Roxb. ex Colebr.

*Boswellia serrata* Roxb. ex Colebr., also known as Indian frankincense or kundur in Unani medicine, belongs to the Burseraceae family. The plant is native to India, widely distributed in dry filly forests of Madhya Pradesh, Rajasthan, Gujarat, Assam, Bihar, Orrisa, and central peninsular regions of Andhra Pradesh. Traditionally the oleo-gum resin of the plant was used as an effective remedy for fevers, cough, bronchitis, asthma, diarrhea, mouth sores, dysentery, ringworm, cardiovascular diseases, skin diseases, blood diseases, inflammatory diseases, and vaginal discharges. The key bioactive component of the plant extract is boswellic acid [40,41,42]. The plant extract and active component, boswellic acid, have proven efficacy in treating adults with asthma, arthritis, and inflammatory bowel disease, as evident by randomized clinical trials, but their clinical effectiveness in pediatrics in these disorders is currently lacking [43,44,45]. A single study conducted by Janssen et al. (2000) evaluated the effectiveness of boswellic acid in the palliative therapy of children with brain tumors [15]. Nineteen children and adolescents (mean age: 8.4 years) with progressive or relapsed brain tumors received a palliative therapy of boswellic acid at a maximum dose of 126 mg/kg/day for a duration of 1 to 26 (median 9) months. Improvement in the general status of patients and neurological symptoms (parses and ataxia) with increased muscular strength, regression of peritumoral edema, and regression of the volume of a tumor cyst were observed in the treatment group.

#### 3.1.5. *Valeriana officinalis* L.

Valeriana officinalis L., commonly known as Valerian, is a flowering plant from the genus Valeriana of the Caprifoliaceae or Valerianaceae family. The plant is native to Asia, Europe, and North America and is included in at least 20 pharmacopeias worldwide. Valerian contains a sesquiterpenoid, valerenic acid as the main constituent [46]. In Traditional medicine, the plant has been used in spasms, cardiac arrhythmias, insomnia, hysteria, hypochondriasis, emotional states, convulsions, digestive problems, and urinary tract infections [46,47].

Müller and Klement investigated the effectiveness of combined valerian and lemon balm in the treatment of children with restlessness and dyssomnia in a multicenter observational study [16]. Nine hundred and eighteen patients were selected for the study and were administered with a maximum of 2 × 2 tablets each day for the period of 4 weeks, where each tablet contained valerian root dry extract (160 mg) and lemon balm extract (80 mg). An improvement in symptoms associated with restlessness and dyssomnia was observed over the 4-week treatment with valerian and lemon balm. Nevertheless, a study performed by Keneddy and colleagues should be taken into account, showing that a lower dose of lemon balm and valerian can be helpful in relieving anxiety but a higher dose might be associated with a slight increase in anxious states [48].

In a randomized double-blind placebo-controlled trial, 30 patients (5–11 years age) pre-diagnosed with ADHD were treated with *V. officinalis* mother tincture (MT) or *V. officinalis* 3X three times a day for 2 weeks, in order to establish the efficacy of homeopathic preparations of *V. officinalis* for this condition [17]. A significant improvement in ADHD symptoms was noted in patients treated with *V. officinalis* MT or 3X in reference to sustained attention, impulsivity, hyperactivity, and anxiety.

#### 3.1.6. *Eschscholzia californica* Cham.

*Eschscholzia californica* Cham. (California poppy) belongs to the Papaveraceae family and is native to North America and Mexico but is now widely cultivated worldwide. California poppy has been traditionally used in algesia, insomnia, anxiety, bed-wetting (especially in children), and incontinence. The plant extract contains a number of alkaloids including protopine, protoberberine, aporphine, pavine- and benzophenanthridine-type alkaloids [49,50,51,52].

*E. californica* is related to another type of poppy and is a very gentle herb for calming the nervous system, which makes it a better alternative for the treatment of pain, restlessness, and insomnia. Unlike poppy plants, it does not contain opiates and thus it is very safe to use in children over 2 years of age [53]. The findings from an in vivo study suggest that *E. californica* possesses anti-anxiety effects at a 25 mg/kg/mouse dose and sedative effects at doses above 100 mg/kg/mouse [54]. A double-blind, randomized, placebo-controlled study showed promising results with a fixed combination of plant extracts (*Crataegus oxyacantha* L. (synonym of Crataegus rhipidophylla Gand.) and *E. californica*) in the treatment of mild to moderate anxiety disorder in adult patients [55]. However, relevant evidence for using California poppy in children with anxiety or sleeplessness is still lacking [56]. Thus, due to the lack of data in children, no recommendation can be made.

#### 3.1.7. *Grindelia robusta* Nutt.

*Grindelia robusta* Nutt. belongs to the Asteraceae family, which is widely cultivated in the Western parts of North America. The plant has been used by Native Americans as an alternative treatment for cough, asthma, and pneumonia [57,58]. Canciani et al. (2014) evaluated the efficacy of pediatric syrup Grintuss^®^ containing saponins, polysaccharides, resins, flavonoids, and sugars derived from *G. robusta*, *Helichrysum italicum* (Roth) G.Don, *Plantago lanceolata* L., and honey, in treating cough in children in multicenter double-blind, placebo-controlled clinical trials [18]. Children with persistent coughs lasting more than 7 days were enrolled and were treated with Grintuss^®^ syrup 4 doses/day, 5 mL each dose for 8 days. The results showed significant anti-tussive effects from treatment with Grintuss^(R)^ syrup when compared with a placebo. Similarly, in a multicenter single-blind randomized trial, a pediatric cough syrup containing specific fractions of polysaccharides, resins, saponins, flavonoids, and sugars derived from *G. robusta*, *H. italicum*, and *P. lanceolata* notably improved nocturnal and daytime cough when used for the treatment of children with acute cough (2–5 years of age), administered at 20 mL/day in three divided doses for four consecutive days [19].

#### 3.1.8. *Malva sylvestris* L.

*Malva sylvestris* L. (common mallow) is an annual or biennial plant from the Malvaceae family, native to Asia, North Africa, and Europe [59,60]. The plant has traditionally been used as an emollient, antitussive, and laxative for a long time [59]. The phytochemical analysis of *M. sylvestris* showed the presence of malvone A, monoterpenes, flavonoids, coumarins, polysaccharides, mucilages, vitamins (vitamins C and E, and beta-carotene), fatty acids (omega-3 and omega-6), sterols, and amino acids [61]. *M. sylvestris* has been added to other botanical extracts in childhood supplements such as Floradix^®^ syrup, to strengthen the immune system and to increase the body’s resistance to infections [62].

The safety and efficacy of a pediatric cough syrup KalobaTUSS^®^, which consists of acacia honey and extracts from *Inula helenium* L., *M. sylvestris*, *Helichrysum stoechas* (L.) Moench and *Plantago major* L. have been evaluated in a randomized controlled trial [20]. One hundred and six children with persistent cough were recruited for the study and were treated with cough syrup or a placebo at four times, 5 mL doses daily for eight days. Children treated with cough syrup showed a significant reduction in the severity and duration of cough as compared to the placebo. As mentioned earlier in this review, children treated with an herbal mixture containing *M. sylvestris* along with other botanical extracts for 5 days showed a significant reduction in the severity of cough and nighttime awakenings [14]. An in vitro study showed amelioration of inflammatory responses with *M. sylvestris* in human oral cells co-infected with *Aggregatibacter actinomycetemcomitans*, due to its dual antimicrobial and anti-inflammatory effects [63]. This could also support a medical application of *M. sylvestris* in periodontal disease, which needs further clinical evaluation in a pediatric population.

#### 3.1.9. *Passiflora incarnate* L.

*Passiflora incarnata* L., referred to as purple passionflower, belongs to the *Passifloraceae* family, widely spread in tropical and warm temperate regions. Traditionally, the plant has been used for the treatment of insomnia, anxiety, cough, sexual dysfunction, convulsion, and cancer [64]. Phytochemical analysis has demonstrated the presence of phenolic compounds, alkaloids, and cyanogenic compounds, with flavonoids being the most common phytoconstituents [65,66].

An herbal triplet including *V. officinalis, Hypericum perforatum L.*, and *P. incarnate* was evaluated for the treatment of nervous agitation in children in a multicenter, prospective, observational study, including 115 children in the age range of 6 to 12 years [21]. Dry extracts from *V. officinalis* (28 mg/tablet), *H. perforatum* (60 mg/tablet), and *P. incarnata* (32 mg/tablet) were administered in tablet form via the oral route. The patients were assessed at baseline, then after 2 weeks and then 4 weeks of treatment. The result showed a distinct improvement in children with attention problems, social withdrawal, and mood troubles (anxiety and depression). Earlier, a double-blind randomized controlled pilot trial showed *P. incarnate* to be an effective treatment for generalized anxiety disorder, albeit taking a long time to show effects. However, the trial demonstrated less job impairment during the day in comparison to oxazepam [67].

Akhondzadeh and colleagues studied the efficacy of *P. incarnate* in the treatment of ADHD in children and adolescents [22]. Thirty-four children with ADHD were recruited for an 8-week double-blind randomized clinical trial and were treated with *P. incarnata* (0.04 mg/kg/day) or methylphenidate (1 mg/kg/day) tablets two times a day. The primary outcome was the Parent and Teacher ADHD rating scale, and the patients were examined at baseline, 14, 28, 42, and 56 days after the start of treatment. No significant differences were observed among the groups treated with *P. incarnata* and methylphenidate. Both treatments were clinically effective in the improvement of ADHD as registered by both parents and teachers. However, *P. incarnata* was observed to be inferior to methylphenidate in decreasing anxiety and nervousness.

#### 3.1.10. *Mentha spicata* L.

*Mentha spicata* L. (spearmint) is a member of the Lamiaceae family and is widely spread in temperate and sub-temperate zones. *M. spicata* has been used as a flavoring, spicing, and medicinal agent since ancient times. The essential oil of *M. spicata* is of primary use as stimulative, stomachic, anti-spasmodic, antiseptic, and diaphoretic. It also possesses therapeutic benefits in flatulence, food poisoning, fever, cold, flu, rheumatism, sinusitis, earaches, stings, and hiccups [68,69,70]. In Pakistan, the leaves of *M. spicata* have been reported for use in children with vomiting, diarrhea, earache, nasal infection, intestinal worms, and hepatitis [71]. However, the evidence available regarding the proper dosage of spearmint in children is insufficient [72].

Fitzgerald et al. (2007) documented a pilot study revealing that Latino children have stronger preferences for the scent of spearmint in comparison to Caucasian children [73]. A blend of aromatherapy essential oils extracted from *M. spicata*, *Mentha piperita* L., *Zingiber officinale* Roscoe, and *Lavandula angustifolia* Mill. in equal proportions was evaluated in the treatment of postoperative nausea and vomiting in children by Kiberd and colleagues [23]. Thirty-nine patients across an age range of 4 to 16 were included in the pilot randomized controlled trial, and children with postoperative nausea were treated with a single dose of saline placebo or aromatherapy. Results showed a small, non-significant improvement of postoperative nausea and vomiting with aromatherapy, and authors recommended the formulation for a large-scale randomized control trial.

#### 3.1.11. *Cuminum cyminum* L.

*Cuminum cyminum* L., commonly known as cumin, belongs to the Apiaceae (Umbelliferae) family, and is native to the Mediterranean region, though it has been cultivated throughout the world including Asia, North Africa, and Central Europe. Cumin seeds are commonly used as spices and in traditional medicines as stomachic, diuretic, stimulant, carminative, abortifacient, and astringent agents [74,75]. The chemical composition of *C. cyminum* consists of anthraquinones, coumarins, flavonoids, alkaloids, glycosides, tannins, proteins, steroids, resins, and saponins [76]. A report found mothers treating digestive problems, gas pains, constipation, nausea, and vomiting with the use of herbal supplement products, including cumin water, fennel, aniseed, mint-lemon, linden teas, and rosemary [31]. However, clinical trials demonstrating the effectiveness of cumin in childhood pathologies are lacking.

#### 3.1.12. *Pimpinella anisum* L.

*Pimpinella anisum* L. (aniseed) is an annual herb from the Apiaceae family, native to the Mediterranean region [77,78]. *P. anisum* L. is used in traditional medicines across different civilizations for the treatment of minor ailments. The seeds are commonly recommended as antiseptics, antimicrobials, antioxidants, anti-inflammatory, digestives, antispasmodics, expectorants, and as estrogenic and diuretic agents [79]. *P. anisum* is a part of Hipp^®^ Mixed Herbal Tea (Hipp) along with *M. chamomilla* and *F. vulgare*, which helps in preventing the formation of gas in the digestive system and relieving gas pains and can be used from the second week onwards [62]. Burgess et al. (2010) conducted a 14-day randomized clinical trial to evaluate the superiority of coconut and anise spray over permethrin 0.43% lotion for head louse infestation [80]. Eighty-five children and 15 adults with head louse infestation were included in the study and were treated with either coconut and anise spray for 15 min and removed with shampoo, or permethrin lotion for 45 min and rinsed with water alone. A more significant effect was noted with the spray (82% cure rate) in comparison to the lotion (42% cure rate).

### 3.2. Bioactive Components

The evidence from clinical trials of supplementation of bioactive food components in children have been summarized in Table 2.

#### 3.2.1. Butyric Acid

Butyric acid (C4:0) is a short-chain fatty acid (SCFA), with an etymology coming from the Greek word for butter. It is the principal source of energy for colonocytes and accounts for 83% of total SCFAs present in the colon, in combination with acetic and propionic acids. Butyrate possesses trophic action to the intestinal walls and stimulates the absorption of sodium and water in the intestine [95,96,97]. Natural sources rich in butyrate include butter, cheese, yogurts, creams, milk powder, and some bakery products [98]. A well-balanced diet comprised of probiotics, dietary fibers, and prebiotics may provide adequate quantities of butyrate to the body, where colonic microbial fermentation of dietary fibers and prebiotics facilitate the production of butyrate [99,100,101]. Oral supplementation of butyrate is a novel approach in the therapeutics of numerous chronic disorders including gastrointestinal (GI) disorders, congenital chloride diarrhea (CLD), and hemoglobinopathies [102].

A case report demonstrated butyrate as an effective treatment for CLD in an 11-year aged boy admitted to hospital due to recurrent abdominal sub-occlusions and chronic watery diarrhea [81]. The effect of butyrate was investigated in CLD in a dose-dependent manner, starting from 50 mg/kg/day administered in two doses for 1 week, and increased by 25 mg/kg/day each consecutive week to a maximum of 100 mg/kg/day for next 12 months. Results yielded a normalization of stool pattern and serum/fecal electrolyte concentration at the dose of 100 mg/kg/day. A pro-absorptive effect was induced by butyrate on Na^+^, Cl^-^, and K^+^ intestinal transport, as demonstrated by rectal dialysis. Furthermore, the butyrate was well tolerated as observed in the 12-month period, with no clinical adverse effects or episodes of dehydration reported.

Butyrate has also been shown to be an effective agent in the treatment of GI inflammatory diseases [103], GI infections [104], and sickle cell disease [105], however, clinical trials in children with these disorders are still lacking.

#### 3.2.2. Probiotics

Probiotics are live microbial feed supplements that confer health benefits when consumed in sufficient quantities, preferably by improving or restoring the gut flora [106]. These generally include *Lactobacillus*, *Bifidobacterium, Saccharomyces* genera, some strains of *Escherichia coli*, and some Gram-positive cocci. They should be nonpathogenic and non-toxic, and can be consumed as gel, paste, powder, liquid, or capsule forms, being able to adhere to gut epithelial tissues and produce SCFAs [107,108,109]. Probiotic supplementation can regulate immunity of the GI mucosa, enhance intestinal epithelial integrity, protect gut barrier disruption, and inhibit the growth and/or activity of pathogenic microbes in the GI. Commercially available probiotic products should contain the optimal number of colony-forming units (CFUs) for each microbial strain above the critical threshold (10^6^ CFU). Though the optimal number of CFUs for each bacterial strain delivered remains unknown, the daily recommended doses of probiotics are in the range of 10^6^ to 10^9^ [110]. The ESPGHAN Committee on Nutrition recommends the addition of probiotic strains to dietetic products designed for infants as health-promoting or disease-preventing agents, however, the Committee report stressed the marketing of nutritional formulas with probiotics only where a full evaluation of the benefits and safety of selected probiotic strains had been performed [111].

A meta-analysis of clinical trials on probiotic administration in early life atopy and asthma demonstrated a reduction in the risk of atopic sensitization and total IgE levels in children supplemented with prenatal and/or early-life probiotics; conversely, the risk of asthma and wheezing was found to not be reduced [112]. Milk containing probiotic *Lactobacillus rhamnosus* GG was evaluated in respiratory illness in children in a randomized clinical trial [82]. Five hundred and twenty-three children aged 2 to 6 years were recruited for the study and were supplemented either with normal milk or the same milk with added *L. rhamnosus* GG taken with three daily meals for 28 days. The data analysis showed consumption of GG reduced the occurrence of respiratory illness in children attending daycare centers in the completed cases subgroup, but not in the total population.

Grandy et al. (2010) observed the effects of probiotics in the treatment of acute rotavirus diarrhea in a randomized double-blind controlled clinical trial [83]. Bolivian children aged 1 to 23 months, hospitalized for acute rotavirus diarrhea, were treated with one of the following treatment regimens: oral rehydration therapy plus placebo, oral rehydration therapy plus *Saccharomyces boulardii*, oral rehydration therapy plus probiotic complex (*Lactobacillus rhamnosus*, *Lactobacillus acidophilus*, *Bifidobacterium longum*, and *Saccharomyces boulardii*). Sixty-four subjects finished the protocol, and the analysis of the results revealed a significant decrease in the median duration of diarrhea, vomiting, and fewer in the probiotic treatment group when compared with the placebo, while no effect on the duration of hospitalization was seen.

The role of probiotics in children with autism spectrum disorder (ASD) was evaluated by Shaaban and colleagues in a prospective, open-label study [84]. Thirty autistic children aged 5 to 9 years were supplemented for 3 months with a probiotic formula containing 100 × 10^6^ CFU/g of three probiotic strains (*L. rhamnosus*, *L. acidophilus*, and *B. longum*). The quantitative polymerase chain reaction (q-PCR) of stool samples showed an increase in the colony units of *Lactobacilli* and *Bifidobacteria* levels, with a significant decrease in body weight and improvement in the severity of autism and GI symptoms, compared to the baseline results.

#### 3.2.3. Amino Acids

Amino acid-based formulas are designed to supplement infants and children with severe cow milk allergies and are intended to provide protein in the form of amino acids with no peptide [113]. It has been hypothesized that child stunting may be associated with inadequate dietary intake of essential amino acids (tryptophan, leucine, isoleucine, threonine, valine, methionine, phenylalanine, lysine, and histidine), conditionally essential amino acids (glutamine, glycine, and arginine), and choline [114]. Bala and colleagues showed a direct relationship of variation in plasma concentration of amino acids (phosphoethanolamine, histidine, 3-methyl histidine carnosine, homocysteine, cysteine, methionine, cystathionine, and threonine) and the phenylalanine/tyrosine ratio with pathogenesis of ASD [115]. In 1996, a panel of pediatric neurologists recommended the oral use of amino acid derivative L-carnitine (100 mg/kg/day or a maximum dose of 2 g/day) in childhood epilepsy, carnitine deficiency syndromes, symptomatic valproate associated hyperammonemia, liver or renal toxicities, and premature infants receiving parenteral nutrition [116].

Jordan et al. (2016) evaluated the effects of glutamine and standard parenteral nutrition on heat shock protein 70 (HSP 70) and IL-6 and IL-10 in a randomized clinical trial in critically ill patients [85]. Children with ages ranging from 1 month to 14 years, diagnosed with severe sepsis or post major surgery and requiring parenteral nutrition for at least 5 days, were included in the study and were randomized to receive either standard parenteral nutrition (*n* = 49) or standard parenteral nutrition plus glutamine supplementation (*n* = 49). It was noted that glutamine supplementation can maintain high HSP 70 levels for a longer time, while the effect on IL-6 was not significant, and no effect of glutamine was seen on IL-10.

A randomized trial showed an improvement in erythrocyte glutathione (GSH) synthesis rate in children with severe edematous malnutrition provided with cysteine supplementation [86]. Erytrocyte cysteine, GSH concentrations, fractional and absolute GSH, were measured in two groups of 16 edematous malnourished children (age: 6–18 months) at three times following hospital admission: at 2 days (period 1), at 11 days, when they were malnourished and infected (period 2), at 50 days, when they malnourished but cleared from infection (period 3), and when they recovered. Children were supplemented either with 0.5 mmol/kg/day N-acetylcysteine (NAC group) or alanine (control group) immediately after period 1 and continued until recovery. The concentration and absolute synthesis of GSH increased significantly from period 1 to period 2 in the NAC group. The result suggested that cysteine supplementation may restore GSH synthesis rate and concentration during the early phases of treatment.

#### 3.2.4. Vitamin D

Vitamin D is a fat-soluble vitamin that can be obtained either from dietary sources (oily fish, liver, organ meats, and egg yolks) or synthesized in the skin in the presence of ultraviolet-B (UV-B) radiation [117]. Children aged 2–8 years mostly have adequate dietary consumption of micronutrients including vitamin D, while children older than 8 years may require additional intake of micronutrients which can be fulfilled by the addition of dietary supplements containing micronutrients [118]. For the treatment of vitamin D deficiency states, the American Academy of Pediatrics (AAP) recommends taking a high dose vitamin D regimen for an initial 2 to 3 months, i.e., 1000 IU/day in neonates (up to 1 month of age), 1000–5000 IU/day in infants (1 to 12 months), and 5000 IU/day in children (above 12 months) [119].

A meta-analysis report of small-scale randomized clinical trials has demonstrated an improvement of atopic disease including asthma in children supplemented with vitamin D [120]. Low vitamin D levels in children may worsen asthmatic control and lung function and may contribute to therapy-resistant asthma [121]. Prenatal supplementation of vitamin D can reduce the risk of asthma and recurrent wheezing in offspring, as evidenced by randomized clinical trials [122]. A double-blind, randomized clinical trial was conducted in Mongolia to evaluate the effectiveness of vitamin D in acute respiratory infection in school children [87]. Seven hundred and forty-four school children were randomly assigned to receive different treatments in winter (January–March), 247 of which were assigned to daily consumption of unfortified regular milk (control, *n* = 104) or milk fortified with 300 IU of vitamin D3 (*n* = 143). The baseline vitamin D3 level was 7 ng/mL, while at the end of the study the vitamin D3 level was found to be significantly higher in the group of children receiving fortified milk (19 ng/mL), as compared to the control group (7 ng/mL). A significantly low rate of acute respiratory infections was found during the study period in children receiving vitamin D3.

As demonstrated by a randomized controlled trial, vitamin D supplementation could reduce the occurrence of pneumonia episodes [88]. The study was carried out in Kabul by recruiting 453 children aged 1–36 months diagnosed with pneumonia. A single dose of 100,000 IU Vitamin D3 in pharmaceutical form (oral drops) (*n* = 224) or placebo (*n* = 229) was added to routine treatment of patients. There was no significant difference in the mean number of days to recovery between the vitamin D3 and placebo groups, though the risk of a repeat episode of pneumonia in children receiving vitamin D3 was significantly lower compared to the placebo group.

The effect of higher (1200 IU/day) and standard (400 IU/day) dosages of vitamin D3 in pharmaceutical form was investigated by Rosendahl et al. on bone health and infection in healthy infants [89]. A randomized clinical trial was conducted by including 975 healthy infants, carried out from January 2013–June 2014, while the final follow-up was conducted in May 2016 to analyze the results. Children aged 2 weeks to 24 months were randomly assigned a higher dose of vitamin D3 (*n* = 486) or a standard dose (*n* = 489), where the primary outcomes were bone strength and incidence of reported infections. A standard dose of vitamin D3 (400 IU/day) was found adequate to maintain vitamin D sufficiency in children younger than 2 years, with increased bone strength and reduced rates of infections. The higher dose (1200 IU/day) did not show any additional benefits over the standard dose in terms of bone health and incidence of infections.

#### 3.2.5. Zinc

Zinc (Zn) is the second most abundant transition metal in the body following iron (Fe). It is one of the essential minerals that fulfill basic physiological needs of the body, such as normal growth and development, metabolism, and maintenance of cell integrity and functionality [123,124,125]. A literature review strongly supported the contribution of Zn deficiency in children to faltering growth, where studies showed that even mild to moderate Zn deficiency affects growth in children [126].

Lind et al. (2004) conducted a randomized clinical trial by recruiting 680 Indonesian children (6–12 months age) to investigate the potential role of Zn and Fe on growth and development [90]. Children were randomly assigned to receive placebo, 10 mg Fe, 10 mg Zn, or a combination of Fe and Zn (10 mg each) for 12 months. It was noted that single supplementation with Fe significantly improved growth and psychomotor development while single supplementation with Zn significantly improved growth, but combined supplementation with Fe and Zn showed no additional benefits.

A pooled analysis of randomized controlled trials showed a reduction in the rates of diarrhea and pneumonia with Zn supplementation in children in developing countries [127]. A double-blind, placebo-controlled, randomized clinical trial revealed an improvement in cholera in children in Bangladesh [91]. One hundred and seventy-nine children aged 3–4 years with watery diarrhea, and who tested positive for *Vibrio cholerae,* were randomly assigned to receive 30 mg/day elemental Zn (*n* = 90) or placebo (*n* = 89) until recovery. Additionally, each patient received erythromycin suspension (12.5 mg/kg) every 6 h for three days. Eighty-two patients in each group completed the study and the Zn supplemented patients showed faster recovery and 12% shorter duration of diarrhea than placebo, with 11% less stool output.

Acevedo-Murillo et al. (2019) investigated the therapeutic effects of Zn supplements in children younger than 5 years old with pneumonia [92]. One hundred and three children were recruited in a randomized controlled clinical trial and received Zn sulfate (10 mg for children younger than 1 year age and 20 mg for children older than 1 year age) or a placebo. The analysis of different parameters revealed an improvement in the patients’ clinical status, respiratory rate, and oxygen saturation with Zn supplementation in fewer hours. Moreover, an increase in blood levels of interferon (IFNγ) and interleukin (IL-2) was observed in the Zn-treated group. Conversely, a community-based, cluster-randomized clinical trial conducted by Tielsch et al. in Nepal from October 2001 to January 2006, illustrated no significant differences between Zn supplementation and placebo intake on a child’s overall mortality [128]. Furthermore, it is wise to mention here that supplement use in children may also increase the prevalence of intake above the upper limits of several minerals, with Zn among the most notable components [118].

#### 3.2.6. Choline

Choline is an essential nutrient that is required for the maintenance of physiological health and should be consumed in the daily diet [129]. Whey protein, eggs, fishes, beef, beans, pork, chicken, milk, wheat germ, and almonds are some foods rich in choline [130]. An adequate intake of choline in prenatal and early postnatal periods could play an important role in brain and memory development, enhancing cognitive performance in childhood, and preventing childhood neurological diseases (Down syndrome, Rett syndrome, and ASD) [131,132]. An intake of infant nutritional formulas containing choline is recommended where infants and children are unable to receive a sufficient amount of choline. The guidelines suggest a minimum concentration of choline content in infant formulas of 7 mg/100 kCal and a maximum concentration of 30–50 mg/100 kCal [133].

Schall et al. (2016) studied the therapeutic effects of choline supplementation in children with cystic fibrosis in randomized placebo-controlled trials [93]. One hundred and ten children (age: 5 to 17.9 years) with cystic fibrosis and pancreatic insufficiency were treated with choline-rich structured lipid (LYMX-SORB™ or LXS) for 12 months in a dose range equivalent to a choline concentration of 591–887 mg/day. LXS powder, comprised of lysophosphatidylcholine, monoglycerides, and fatty acids in a molar ratio of 1:4:2, was mixed with participant-selected foods and beverages for ease of administration. The muscle and plasma concentration of choline was increased in the LXS treated group, accompanied by improved dietary fat absorption, suggesting a potential role for choline-containing supplements in improving nutritional and growth status in patients with cystic fibrosis and pancreatic insufficiency. Moreover, choline supplementation resulted in increased plasma concentration of glycine, with decreased threonine, valine, histidine, and total branched-chain amino acid levels after 12 months of treatment.

Wozniak and colleagues evaluated the effects of choline supplementation in children with fetal alcohol spectrum disorder in a randomized double-blind placebo-controlled trial [94]. Sixty children aged 2.5–5 years were treated either with 500 mg of choline or a placebo daily for 9 months. Mullen Scales of Early Learning was taken as the primary outcome, while elicited imitation memory paradigm was used as a secondary measure. A significant improvement in primary and secondary measures of memory was observed in the choline-treated group.

## 4. Safety Aspects of Childhood Supplementation

When compared to drugs, the current studies available on the safety of botanical extracts in childhood supplementation are limited, however, their use is more frequent for the treatment of minor ailments due to their perceived efficacy, safety, and often low cost. On the other hand, children are more prone to certain risks with dietary supplementation such as incorrect dosing, side effects, drug–supplement interactions, and allergic reactions [134,135]. The quality of supplements and lack of standardization are obstacles in making recommendations for the use of botanical extracts and bioactive food components for the prevention or management of symptoms of mild ailments in children and adolescents. Mislabeling, misidentification, adulteration with other herbs or pharmaceuticals, and contamination with heavy metals, herbicides, and pesticides could lead to serious adverse events in pediatrics [136]. A recent study conducted by Saper and colleagues showed that about 20% of herbal products were contaminated with heavy metals including lead, mercury, and arsenic, where 50% of those products were marketed for use in childhood supplementation [137]. There is always a risk of drug–supplement interactions, as parents do not report the use of supplements to the primary healthcare professional of their children. A survey report showed that only 45% of parents report the use of botanical supplements to the primary healthcare provider of children [134].

For instance, *M. chamomilla* is a potential vehicle of *Clostridium botulinum* spores and thus ingestion of chamomile tea could be a possible risk for infant botulism [138]. A 13-old boy was diagnosed with hepatic failure, where liver biopsy showed more than 90% necrosis of hepatocytes, and the most likely cause identified was Euphytose (a formulation mixture of *V. officinalis*, *Crataegus oxyacantha*, *Ballota nigra*, *P. incarnata*, and *Cola nitida*) [139]. Probiotics seem to be safe for use across all premature ages (infants, children, and adolescents), as no serious adverse effects have been noted with the supplementation of *L. plantarum*, *L. reuteri*, and *B. lactis* [140,141], however, theoretical concerns regarding the safety of probiotic species still exist such as bacteremia, endocarditis, toxic effects on GI tract, and transfer of antibiotic resistance in GI flora [142]. Children should be closely observed with vitamin D supplementation because of the increased risk of associated hypercalcemia and nephrocalcinosis [143]. A study reported hypercalcemia in infants supplemented with a single dose of 300,000 IU [144].

## 5. Conclusions

The supplementation of herbal products or bioactive components reported above in children is relatively safe, based on both their traditional use and the clinical trials, with no serious adverse event being reported. However, studies highlighting the safety issues of childhood supplementation are limited and above all, there are no studies specifically aimed at assessing safety in children. Thus, time is needed to expand the literature data covering both the efficacy and the safety of childhood supplementation with botanical extracts and bioactive food components, especially regarding the dosage, the adequate method of intake to avoid interaction with drugs, and other foods or food components.

Though no specific recommendation can be made at this point towards the use of childhood supplements, the use of botanical extracts and bioactive components alone or in combination with conventional therapies might be considered on an individual basis to manage various ailments.

## Figures and Tables

**Table 1:** Evidence of botanical extract supplementation in children from clinical trials.

Botanical Extract	Study Design	Intervention	Main Results	Reference
Compound herbal preparation (*M. officinalis,* *W. somnifera*, *B. monnieri*, *A. platensis*, and *C. asiatica*)	Randomized controlled trial, 120 children with ADHD (mean age: 9.82 years for treatment group and 9.36 years for control group) were recruited.	Three mL of compound herbal preparation 3 times a day in 50–60 mL water.	Significant improvement of TOVA scores, attention, cognition, and impulse control in intervention group.	[10]
*M. officinalis* and *P. decandra*	Crossover randomized triple-blinded controlled trial, 52 children with sleep bruxism with mean age of 6.62 years were selected.	The study included 4 phases of 30-day treatment (placebo, *M. officinalis* 12 c, *P. decandra* 12c and *M. officinalis* 12c + *P. decandra* 12c) with a washout period of 15 days between treatments.	Significant decrease in VAS in *M. officinalis* treated phase. No improvement of results was seen in combination of *M. officinalis* with *P. decandra*.	[11]
*F. vulgare*	Double blind, placebo-controlled study, 125 infants with 2–12 weeks of age, diagnosed with infantile colic were selected for the trial.	A mixture of 0.1% of *F. vulgare* oil emulsion and 0.4% polysorbate in water. Five to twenty milliliters of mixture administered 4 times a day before meal at a maximum dose of 12 mL/kg/day.	Significant recovery of the colic symptoms in *F. vulgare* treated group.	[12]
*M. chamomilla* and *M. officinalis*	Multicenter, randomized controlled trial, Children with infantile colic were recruited.	Patients were treated with mixture of *M. chamomilla*, *M. officinalis* and tyndallized *Lactobacillus acidophilus* HA122 or *Lactobacillus reuteri* DSM 17,938 for 28 days.	One hundred and seventy-six children completed the study. The symptoms of infantile colic relieved with a significant decrease in mean daily crying in both groups.	[13]
Herbal mixture of *M. chamomilla*, *A. officinalis*, *H. officinalis*, *M. sylvestris*, *A. capillus-veneris*, *Z. jujube*, and *G. glabra*	Double-blind randomized clinical trial, 46 children aged 7–12 years old diagnosed with intermittent asthma were selected.	Children were treated with herbal mixture (5 mL three times a day) or placebo for 5 days.	Significant reduction in the severity of cough and nighttime awakenings in the treatment group. No improvement of wheezing, respiratory distress, tachypnea, peak expiratory flow rate, asthma exacerbations, outpatient visits, oral administration of prednisone or β-agonists and hospitalization.	[14]
Boswellic acid (*B. serrata*)	Nineteen children and adolescents (mean age of 8.4 years) with progressive or relapsed brain tumors were selected for trial.	Patients received boswellic acid at a maximum dose of 126 mg/kg/day for duration of 1–26 months (median 9 months).	Improvement of general status of patients and neurological symptoms (parses and ataxia), increased muscular strength, regression of peritumoral edema and regression of the volume of a tumor cyst.	[15]
*V. officinalis* and *M. officinalis*	Multicenter observational study, 918 children with restlessness and dyssomnia were recruited for the study.	Each patient received a maximum of 2 × 2 tablets per day for 4 weeks, where each tablet contains valerian root dry extract (160 mg) and lemon balm extract (80 mg).	Improvement of symptoms associated with restlessness and dyssomnia in intervention group.	[16]
*V. officinalis*	Randomized double-blind placebo-controlled trial, 30 children with ADHD (age: 5–11 years) were selected.	Patients were treated with *V. officinalis* mother tincture (MT) or *V. officinalis* 3X three times a day for 2 weeks.	A significant improvement in ADHD symptoms in patients treated with *V. officinalis* MT or 3X in reference to sustained attention, impulsivity, hyperactivity and anxiety.	[17]
Pediatric syrup Grintuss ^®^ (*G. robusta*, *H. italicum*, *P. lanceolata*, and honey)	Double-blind, randomized, placebo-controlled trial, 102 children aged 3–6 years, with persistent cough for at least 7 days up to 3 weeks and not treated with any antitussive agent were recruited.	Patients were treated with placebo (*n* = 51) or Grintuss ^®^ syrup (*n* = 51) 4 doses/day, 5 mL each dose for 8 days.	Significant improvement in daytime and night-time cough scores.	[18]
Polysaccharide-resin-honey (PRH)-based cough syrup (*G. robusta*, *H. italicum* and *P. lanceolata*)	Randomized, single-blind multicenter study, 150 children aged 2–5 years with upper respiratory tract infection, nocturnal and daytime cough and illness duration of ≤ 7 days were participated.	Patients were treated with PRH cough syrup (20 mL/day) or carbocysteine based syrup (control, 25 mg/kg/day) in three divided doses for 3 consecutive days.	PRH cough syrup showed more rapid and greater improvement in all clinical cough symptoms measured compared to carbocysteine based syrup.	[19]
KalobaTUSS ^®^ pediatric cough syrup (Acacia honey, *I. helenium*, *M. sylvestris*, *H. stoechas*, and *P. major*)	Randomized double-blind, placebo-controlled trial, 106 children with persistent cough are recruited in the study.	Patients were treated with cough syrup or placebo 4 doses daily, 5 mL each for 8 days.	Cough syrup significantly reduces the severity and duration of cough as compared to placebo.	[20]
Herbal triplet (*V. officinalis*, *H. perforatum* and *P. incarnata*)	Multicenter, prospective, observational study, 115 children aged 6–12 years with history of nervousness and agitation (including agitated depression) due to affective disorders were selected for the study.	Dry extract of herbal triplet administered in tablet form via oral route, containing *V. officinalis* (28 mg/tablet), *H. perforatum* (60 mg/tablet) and *P. incarnate* (32 mg/tablet). Patients were accessed at baseline, after 2 weeks of treatment and then after 4 weeks of treatment.	Herbal triplet showed a distinct improvement in children with attention problems, social withdrawal, and mood troubles (anxiety and depression).	[21]
*P. incarnata*	Double-blind randomized clinical trial, 34 children with ADHD were recruited in an 8-week clinical trial.	Children were treated with *P. incarnata* (0.04 mg/kg/day) or methylphenidate (control, 1 mg/kg/day) tablets, two times a day. The patients were examined at baseline and14, 28, 42, and 56 days after the start of treatment.	Both groups were clinically effective in the improvement of ADHD. However, *P. incarnata* was inferior to methylphenidate in decreasing anxiety and nervousness.	[22]
Aromatherapy essential oils (*M. spicata*, *M. piperita*, *Z. officinale*, and *L. angustifolia*)	Pilot randomized controlled trial, 39 patients with age range of 4–16 years with postoperative nausea and vomiting were selected for the trial.	Children were treated with a single placebo or aromatherapy.	Non-significant improvement of postoperative nausea and vomiting with aromatherapy. Though the preparation has been recommended for large-scale randomized clinical trials.	[23]

Attention deficit hyperactivity disorder (ADHD), test of variables of attention (TOVA), visual analogue scale (VAS).

**Table 2:** Evidence of bioactive food supplementation in children from clinical trials.

Bioactive Food Components	Study Design	Intervention	Main Results	Reference
Butyric acid	Case study, 11-year-old boy with CLD, admitted to hospital because of recurrent abdominal sub-occlusions and chronic watery diarrhea.	The patient was treated with butyric acid in dose of 50 mg/kg/day administered in 2 doses for 1 week, which increased gradually in increments of 25 mg/kg/day every consecutive week to a maximum dose of 100 mg/kg/day for the next 12 months.	The normalization of stool pattern and serum/fecal electrolytes concentration with 100 mg/kg/day was observed with a dose of 100 mg/kg/day. Rectal dialysis showed induced pro-absorptive effects induced by butyrate on Na^+^, Cl^−^, and K^+^ intestinal transport.	[81]
Probiotic supplement	Randomized clinical trial, 523 children aged 2–6 years attending day care centers were recruited in the study to evaluate the effects of probiotic supplementation in respiratory illnesses.	Children were supplemented with normal milk or milk containing probiotic *Lactobacillus rhamnosus* GG on 3 daily meals for 28 days.	The probiotic supplementation showed a reduced occurrence of respiratory illness in children attending daycare centers.	[82]
Probiotic complex (*L. rhamnosus*, *L. acidophilus*, *B. longum*, and *S. boulardii*)	Randomized double-blind controlled clinical trial, Children aged 1–23 months hospitalized with acute rotavirus diarrhea were selected for the trial.	Patients were treated with oral rehydration therapy plus placebo, oral rehydration therapy plus *S. boulardii* or oral rehydration therapy plus probiotic complex.	Sixty-four cases finished the protocols and were analyzed for results, which showed a significant decrease in median duration of diarrhea, vomiting and fever in probiotics treated groups. Effect of probiotics on duration of hospitalization was neutral.	[83]
Probiotic formula (*L. rhamnosus*, *L. acidophilus*, and *B. longum*)	Prospective, open label study, 30 autistic children aged 5–9 years were selected for the study.	Children were supplemented with probiotic formula containing 100 × 10^6^ CFU/g of three probiotic strains (*L. rhamnosus*, *L. acidophilus*, and *B. longum*).	q-PCR of stool samples showed an increase in the colony units of *Lactobacilli* and *Bifidobacteria* levels, with a significant decrease in body weight and improvement in the severity of autism and GI symptoms, as compared to baseline results.	[84]
Glutamine supplementation	Randomized clinical trial, critically ill children with age range of 1 month to 14 years that were required parenteral nutrition for at least 5 days were recruited in clinical trial to evaluate the effectiveness of glutamine versus standard parenteral nutrition on HSP 70 and interleukins 6 and 10.	Children were treated with glutamine (*n* = 49) or standard parenteral nutrition (*n* = 49).	Glutamine supplementation maintained high HSP 70 levels for longer time. The effect of glutamine was not significant on IL-6 while the effect on IL-10 was neutral.	[85]
Cysteine supplementation	Randomized clinical trial, 16 edematous malnourished children (age: 6–18 months) were selected for study. Erythrocyte cysteine and GSH concentrations, and fractional and absolute GSH synthesis rates were measured 3 times after hospital admission, at 2 days (period 1), 11 days, when they were malnourished and infected (period 2), 50 days, when they malnourished but cleared from infection (period 3) and when they recovered.	Children were supplemented with 0.5 mmol/kg/day N-acetylcysteine (NAC group) or alanine (control group)	The concentration and absolute synthesis of GSH increased significantly from period 1 to period 2 in NAC group.	[86]
Vitamin D	Double-blind, randomized clinical trial, 744 school children were selected for the trial to demonstrate the effectiveness of vitamin D in acute respiratory infections in winter (January–March).	Children consumed unfortified regular milk (control) or milk fortified with vitamin D3 (300 IU).	The vitamin D level in blood considerably increases in children supplemented with fortified milk (from 7 ng/mL to 19 ng/mL). A significantly low rate of acute respiratory infections was found in these children.	[87]
Vitamin D	Randomized controlled trial, 453 children aged 1–36 months, diagnosed with pneumonia were recruited for the trial.	A single dose of 100,000 IU Vitamin D3 oral drops (*n* = 224) or placebo (*n* = 229) was added to routing treatment of patients.	The risk of a repeat episode of pneumonia in children received vitamin D3 was significantly lower. However, no significant difference was seen on the mean number of days to recover between both groups.	[88]
Vitamin D	Randomized clinical trial, 975 healthy infants aged 2 weeks to 24 months were recruited to compare the effects of standard dose (400 IU/day) versus high dose (1200 IU/day) vitamin D on bone strength and infections.	Children were randomized to receive standard dose of vitamin D3 (*n* = 489) or high dose of vitamin D3 (*n* = 486).	A standard dose of vitamin D3 was found adequate to maintain vitamin D sufficiency in children younger than 2 years, with increased bone strength and reduced rate of infections. A higher dose did not show any additional benefits over standard dose.	[89]
Zinc and Iron	Randomized clinical trial, 680 children (6–12 months age) were recruited to investigate the potential role of Zn and Fe on growth and development.	Children received daily placebo, Fe (10 mg), Zn (10 mg) or Fe + Zn (10 mg each) for 12 months.	Supplementation with Fe alone improved growth and psychomotor development. Zn significantly improved growth. Combined Fe and Zn supplementation possessed no additional benefits.	[90]
Zinc	Double blind, placebo-controlled, randomized clinical trial, 179 children aged 3–4 years with watery diarrhea and tested positive for *V. cholera* were selected for the study.	Patients were randomly assigned to receive daily dose of 30 mg/day elemental Zn (*n* = 90) or placebo (*n* = 89) until recovery. Each patient also received erythromycin suspension (12.5 mg/kg) every 6 h for three days.	Eighty-two patients in each group completed the study. Zn supplements showed faster recovery and 12% shorter duration of diarrhea than placebo with 11% less stool output.	[91]
Zinc	Randomized controlled clinical trial, 103 children younger than 5 years, diagnosed with pneumonia were recruited.	Children received Zn sulfate (10 mg children younger than 1 year and 20 mg for children older than 1 year of age).	Zn supplementation improved patient’s clinical status, respiratory rate, oxygen saturation, and increased blood levels of IFNγ and IL-2.	[92]
Choline-rich structured lipid (LYMX-SORB™ or LXS)	Randomized placebo-controlled trial, 110 children (age: 5 to 17.9 years) with cystic fibrosis and pancreatic insufficiency were included in the trial.	Children were treated with LXS, mixed with participant selected foods or beverages for 12 months in a dose range equivalent to a choline concentration of 591–887 mg/day. LXS powder comprised of lysophosphatidylcholine, monoglycerides and fatty acids in a molar ratio of 1:4:2.	The muscle and plasma concentration of choline was increased in LXS-treated group. LXS supplementation improved the dietary fat absorption and, nutritional and growth status.	[93]
Choline	Randomized double-blind placebo-controlled clinical trial, 60 children aged 2.5–5 years with fetal alcohol spectrum disorder were recruited in the trial.	Children were treated with 500 mg choline or placebo daily for 9 months.	Choline supplementation significantly improved the primary and secondary measures of memory.	[94]

Congenital chloride diarrhea (CLD), colony-forming units (CFU), quantitative polymerase chain reaction (q-PCR), heat shock proteins (HSP), glutathione (GSH), Zinc (Zn), iron (Fe), interferon-gamma (IFNγ), interleukin-6 (IL-6), interleukin-10 (IL-10), interleukin-2 (IL-2).
